# Profiles of Teenage Athletes’ Exposure to Violence in Sport: An Analysis of Their Sport Practice, Athletic Behaviors, and Mental Health

**DOI:** 10.1177/08862605221148216

**Published:** 2023-02-07

**Authors:** Isabelle Daignault, Nadine Deslauriers-Varin, Sylvie Parent

**Affiliations:** 1 Université de Montréal, QC, Canada; 2 International Center for Comparative Criminology, Montréal, QC, Canada; 3 International Research Network on Violence and Integrity in Sport, Antwerp, Belgium; 4 Research Chair in Security and Integrity in Sport, Québec, QC, Canada; 5 Université Laval, Québec, QC, Canada; 6 Interdisciplinary Research Center on Intimate Relationship Problems and Sexual Abuse (CRIPCAS), Montréal, QC, Canada

**Keywords:** adolescent victims, mental health and violence, sexual assault, youth violence

## Abstract

Violence in sport is a major social issue generating great interest in research over the last 10 years. Studies to date highlight various forms and manifestations of violence in the lives of teenagers practicing individual or team sports, in competitive and recreational contexts. Although allegations of sexual violence involving coaches most often reach media attention, psychological and physical violence involving teammates, parents, and coaches are also prevalent. While profiles of offenders in the sport context have contributed to a better understanding of the issue, similar profiles need to be elaborated for young victims to delineate varying degrees of risk, adaptation, and needs. Latent class analyses were conducted to empirically identify different patterns of exposure to violence in sport from a sample of 1057 athletes aged 14–17 years. Teenagers participated in an online survey assessing their experiences of violence using the Violence Toward Athletes Questionnaire. Results highlighted three different profiles of victimization in the sport context: (a) a non-victimized profile constituting only 37% of the sample; (b) a profile representing 52% of the sample that is mainly exposed to psychological violence by teammates, coaches, and parents; and (c) a “poly-victimized” profile, representing 10% of the sample, that is exposed to all forms of violence at the hands of various perpetrators (teammates, coaches, and parents). The identified profiles were compared according to different indicators of sport practice, athletic behaviors, and mental health. This study delineates the influence of single and multiple forms of violence and its compound consequences on mental health and sport-related behaviors, thus portraying various degrees of need for tailored prevention and intervention measures.

Sports practice has been associated with several physical and mental health benefits in both children and youth (Mountjoy et al., 2011). In fact, since the end of the 1990s, the United States National Council of Youth Sports (2008) has observed a social trend, indicating that parents increasingly engage their children in sports, particularly from a younger age. However, there is great variability in how the regular practice of a sport may influence one’s life. Whereas for most children and youth, it results in higher self-esteem and a sense of self-competence (Logan & Cuff, 2019), for others, violence and abuse interfere with such positive outcomes (Mountjoy et al., 2016). Research with former and current athletes conducted in European countries (i.e., United Kingdom, Netherlands, Norway) and in Canada reveals that up to 75% (2%−75%) of youth is exposed to interpersonal violence in the practice of their sport (Fasting et al., 2011; Parent & Vaillancourt-Morel, 2020; Vertommen et al., 2016). Although sexual violence has drawn more media and research attention (Vertommen et al., 2017), a growing body of research is documenting the occurrence of multiple forms of violence including psychological and physical violence, neglect, harassment, and bullying in sport (Mountjoy et al., 2016; Vertommen et al., 2018). While the relationship between exposure to single forms of violence and physical and mental health outcomes have been documented in sport (e.g., Ohlert et al., 2019), few studies have examined the issues of co-occurring violence, or being exposed to more than one form of violence in the life of the same athlete (Finkelhor et al., 2007a). A study by Vertommen et al. (2018) documented such co-occurrence of violence in Dutch and Belgian adults (*n* = 4,043) who were involved in organized sport before the age of 18. Their results show that half of those experiencing one form of interpersonal violence also reported being exposed to a second or a third form. They also found that being exposed to more than one form of violence (referred to as poly-victimization) was associated with higher scores of psychological distress, and lower scores of quality of life. Parent et al. (2021) also found that being exposed to a greater number of violence types was associated with more severe symptomatology.

In a different study, Vertommen et al. (2017) explored profiles of perpetrators by questioning victims of interpersonal violence in sport. The profiles were based on the number of perpetrators and their characteristics (e.g., sex, age, and role in the organization). While research has not yet explored profiles in victims, their results outline findings that are relevant to this study. The most prevalent profile of perpetrator of interpersonal violence does not appear to be the male coach, as often represented in the media, but rather male peer athletes. Moreover, results show that “known others” within the sport organization (excluding coaches and peer athletes) are also responsible of interpersonal transgressions. This observation outlines the importance of considering other likely perpetrators of violence in sport, such as spectators (Vertommen et al., 2017) or parents. Findings mostly lead to question whether profiles of victims could describe diversity among athletes, the variety of victimization experiences, in individual or team sport, or in a recreative or competitive contexts. Are certain sport environments presenting higher risks of interpersonal violence exposure at the hands of different individuals (e.g., coaches, peer athletes, administrators, parents)? Identifying profiles of victims exposed to distinct or cumulative circumstances associated with higher risk is of great utility to guide tailored prevention and early intervention measures (Daignault & Hébert, 2009; Martinez-Torteya et al., 2017).

## Risk Factors Associated with Violence Exposure in Sport

If recreational and competitive sports can prevent hopelessness, depression, and suicide in adolescents (Gore et al., 2001; Taliaferro et al., 2008) as well as youth crime and antisocial behaviors (Kelly, 2013), exposure to interpersonal violence can counterbalance these protective effects (Logan & Cuff, 2019). In sport, a growing body of research identified risk factors for victimization at the individual, relational, organizational, and sociocultural levels (Parent & Fortier, 2018). In terms of individual and relational factors, athletes belonging to an ethnic group, who live with a disability or who identify as LGBTQ (e.g., lesbian, gay, bisexual) are at greater risk of experiencing violence in general, and in sport (Mountjoy et al., 2016) and psychological violence in particular (Denison & Kitchen, 2015; Vertommen et al., 2015). Regarding gender and age, findings vary, depending on the form of violence, the context (individual/team sports), and the perpetrators involved (i.e., coaches, peer athletes). While women are generally more likely to report sexual violence, men report physical violence more frequently (Alexander et al., 2011; Vertommen et al., 2016). More recently, Parent and Vaillancourt-Morel (2020) observed no gender differences for sexual violence, independently of the perpetrator, but showed that female athletes are more likely to be exposed to psychological violence than male athletes.

Research has more consistently documented certain contexts of sports practice that are associated with higher risk of violence exposure; notably the practice of sports initiations and hierarchy intimidation or hazing (Mountjoy et al., 2015) that are more common to team sports. A constant proximity in the coach–athlete relationship (Parent & Fortier, 2018), more common for individual sports, and the intensity and high demands of competitive sports practice also appear to create contexts more prone to violence tolerance and exposure (Mountjoy et al., 2016). As summarized by Parent and Fortier (2018), elite athletes can be particularly at risk, as they are more likely to practice their sport with intensity, in a context of distancing from their family or source of social support, combined with a constant proximity with an authority figure (a coach) that can exert a certain control on the athlete (weight/diet, choice of equipment, sleep/training, social relationships) (Cense & Brackenridge, 2001). Many have also begun practicing their sport at an early age. Malina (2010) hypothesizes that early sports specialization can make athletes more vulnerable to violence exposure; as it has been described as a context of high demands, social isolation, overdependence, and manipulation; often leading to frequent physical injuries and athlete burnout. While risk factors have mainly been associated with single forms of violence, such risks need to be studied in relation to the athlete’s entire experience.

## The Health Cost of Violence Exposure in Sport

Teenagers exposed to interpersonal violence in sport present mental health issue that are classically associated with exposure to single or multiple forms of interpersonal violence (Finkelhor et al., 2007b), including post-traumatic stress disorder (PTSD) and many other indicators of psychological distress such as anxiety, depression, social isolation, and self-harm (Mountjoy et al., 2016; Parent et al., 2021; Vertommen et al., 2018). Based on the consequences associated with violence exposure in the general population, self-injury, disordered eating, and suicide attempts are also of concern for athletes (Mountjoy et al., 2016). Moreover, as discussed by Roberts et al. (2020), the culture of sports performance often favors certain athletic physical features (e.g., skinny, tall) as well as values of compromise, pain endurance, and persistence despite all challenges. Such a climate may render athletes vulnerable to adopt health compromising behaviors such as doping, problematic weight management techniques, eating disorders and self-harm, either in the form of over-training or training and competing with pain or even with injury (Boudreault et al., 2021; Mountjoy et al., 2016). Self-injury and compromising athletic behaviors can also affect sports practice and lead to poor mental and physical health, impaired performance, and dropout (Mountjoy et al., 2016). While the presence of a single form of violence can lead to long-term consequences, multiple exposure is thought to potentiate this effect (Hamby et al., 2014; Ohlert et al., 2019; Vertommen et al., 2018), one of the hypothesized processes being a neurobiological reaction to a constant solicitation of the stress response (Anda et al., 2006). Current state of knowledge therefore reveals a need to further our understanding of the cost of violence exposure in sport by exploring risk and consequences for children and teenage athletes in relation to a representation of the entire athlete experience, including all forms of exposure to violence. Conceptually, such a systemic stance to violence exposure may contribute to improving studies methodologically by making use of larger and diversified samples of athletes practicing sports in a large variety of contexts (Parent & Fortier, 2018).

## Aim of the Study

In contrast to past research focusing on adults’ retrospective accounts of exposure to single forms of violence, this study asks teenagers to unveil a more recent account of co-occurring violence in various sports, while assessing related physical and mental health outcomes. In this field, the common variable-centered approach has contributed to a large-scale recognition of the matter of violence in sport. Yet, this approach often groups together victimized individuals with extremely different experiences and exposed to various contexts, levels of risk, consequences, and needs. This study adopts a person-oriented/profile approach, which in comparison, will allow documentation of the variability among victims, various contexts representing risk, concomitant forms of violence exposure, as well as the different consequences experienced by the same individual (von Eye et al., 2015). The overall objective of this study is to describe the variety of violence exposures among teenage athletes. The specific objectives are to (1) identify distinct profiles of violence exposure in sport among young athletes and (2) determine how profiles of violence are distinct in terms of risk of exposure while accounting for sociodemographics, characteristics of sports participation, athletic behaviors, and mental health.

## Methods

### Participants and Procedures

This study was approved by the accountable University Research Ethics Board. A convenience sample of 1259 French-Canadian athletes, aged 14–17 years and participating in an organized sport (playing within a league, club or sports team with organized training and competition) at the time of the study, was recruited to participate in an online study assessing their experiences in sport. Participants were recruited on a voluntary basis through online advertisements on social media, email lists of sports partners, as well as flyers distributed in sports competitions. Interested participants accessed an anonymous survey through a hyperlink hosted by a secured online survey software, *Qualtrics*, where they electronically signed a consent form before starting the questionnaire. The completion time ranged from 30 to 45 minutes. Among the 1,259 youth that completed the survey, 1,057 (83.8%) were included in this study as they completed all measures. Sociodemographic characteristics of the sample are presented in Table 1. The final sample consisted of 764 girls (72.3%) and 292 boys (27.7%), with a mean age of 15.29 years (standard deviation = 1.07). The sports practiced varied largely, with soccer (21.0%, *n* = 222), volleyball (13.0%, *n* = 137), and swimming (10.8%, *n* = 114) being the most common.

**Table 1.** Descriptive Information About the Study Variables for the Entire Sample.

	% (*n*)
Sociodemographic	
Age (*n* = 1,057)	15.13 (SD = 1.05)
Gender (*n* = 1,057)	
Boy	32.3 (292)
Girl	67.6 (764)
Intersex	0.1 (1)
Sexual orientation, preference (*n* = 1,057)	
Only/mostly heterosexual	91.8 (970)
Only/mostly homosexual	1.8 (19)
Bisexual	1.4 (15)
Asexual	0.2 (2)
Unsure (questioning)	2.2 (23)
Never thought about it, non-specified	2.6 (27)
Ethnic or cultural background	
Canadian	100 (1,057)
Autochthones, First Nation	1.1 (12)
Latin America	1.1 (12)
Afro American	0.4 (4)
Africa	1.6 (16)
Asia	1.7 (18)
Europe and other	6.3 (64)
Presenting a handicap	1.1 (12)
Victimization	
Psychological—Yes	
By teammate (*n* = 1,057)	62.5 (661)
By coach (*n* = 1,042)	68.3 (712)
By parents (*n* = 993)	44.2 (439)
Sexual—Yes	
By Teammate (*n* = 1,057)	22.7 (240)
By coach (*n* = 1,014)	12.1 (123)
Physical—Yes	
By teammate (*n* = 1,057)	18.5 (196)
By coach (*n* = 1,054)	14.5 (153)
By parents (*n* = 1,009)	8.0 (81)
Sport practice	
Sport type (*n* = 1,050)	
Individual	42.6 (447)
Team	53.1 (558)
Both	4.2 (45)
Sport level (*n* = 1,030)	
Local/Regional/Interregional	26.6 (276)
Provincial	47.2 (490)
National/International	26.3 (273)
Weekly hours of practice (*n* = 1,046)	
Less than 5 hours	14.2 (149)
6–10 hours	37.7 (394)
11–15 hours	26.5 (277)
16 and up	21.6 (226)
Early sport specialization—Yes (*n* = 1,057)	24.3 (257)
Training away from parents—Yes (*n* = 1,029)	6.7 (69)
Serious injury—Yes (*n* = 977)	73.7 (720)
Athletic behaviors	
Extreme weight-control behaviors—Yes (*n* = 978)	16.8 (164)
Non-suicidal self-injury—Yes (*n* = 978)	16.6 (162)
Practice despite injuries—Yes (*n* = 978)	55.7 (545)
Mental health	
Self-esteem (*n* = 969)	
Normal	65.1 (631)
Low	34.9 (338)
Psychological distress (*n* = 965)	
Low	44.9 (433)
Moderate	24.5 (236)
Severe	30.7 (296)
PTSD symptoms—Yes (*n* = 958)	37.1 (355)

PTSD = post-traumatic stress disorder; SD = standard deviation.

### Measures

#### Latent class analysis models

As part of the online survey, the *Violence Toward Athletes Questionnaire* (VTAQ; hidden for submission) was used to assess self-reported experiences of interpersonal violence against children and adolescents in sport. The VTAQ consists of 70 items related to a specific category of perpetrator, namely VTAQ-A (violence from peer athletes, 9 items), VTAQ-C (violence from coaches, 36 items), and VTAQ-P (violence from parents, 25 items). The athlete and coach sections of the questionnaire assess three forms of violence: psychological and neglect, physical, and sexual, while the parent section excludes sexual violence. Items are rated on a four-point Likert scale measuring the frequency with which various events took place in the sport context, where 0 = *never*; 1 = *rarely, 1–2 times;* 2 = *sometimes, 3–10 times*; and 3 = *often, more than 10 times.* The scale showed high internal consistency in our sample with ordinal Cronbach’s α between .79 and .98.

Sexual violence was defined as “a sexual act that is committed or attempted by another person without freely given consent by the victim or against someone who is unable to consent or refuse” (Basile et al., 2014, p. 11). Items of sexual violence included sexual harassment (e.g., offensive sexual remarks on sexual life, on the body), sexual assault (e.g., unwanted sexual contacts), contact and non-contact child sexual abuse (e.g., voyeurism, exposure to pornography, sexual intercourse). Physical violence was defined as any action of a physical nature that compromises or threatens the integrity, physical or psychological well-being of a person (Clément & Dufour, 2009). Items included hitting, pushing, or shaking an athlete. Psychological violence was defined as acts which include restriction of movement, patterns of belittling, denigrating, scapegoating, threatening, scaring, discriminating, ridiculing, or other non-physical forms of hostile treatment or rejection (World Health Organization [WHO], 1999). Items included behaviors that promote the corruption, exploitation and adoption of destructive, antisocial or unhealthy behaviors of a young athlete in the context of sport in relation to a person in a position authority (e.g., force an athlete to train injured despite medical advice, force an athlete to commit acts of violence). Violence items related to neglect from coaches and parents were also included in this category. For this study, eight dichotomous variables (0 = No; 1 = Yes) related to the type of interpersonal violence (i.e., sexual, psychological, and physical) that the participant might have been exposed to at least once, in a sport context, as well as the perpetrator (i.e., coach, parent, peer) of such violence, were used to identify profiles of victimization among teenagers (see Table 1) ^1^.

#### Description and external validity of latent class analysis model

To further describe the identified latent class analysis (LCA) profiles, 14 variables related to the sociodemographic, sports practice, athletic behaviors, and mental health characteristics of the participants were used (see Table 1 for the descriptive statistics for the entire sample).

#### Sociodemographics

Variables related to gender/gender identification (0 = male; 1 = female, intersex), age of the participants at the time of the survey, sexual orientation preference, ethnic/cultural background, and youth presenting a handicap were initially considered. Based on prevalence estimates, only gender (male and female) and age were maintained for further analyses.

#### Sports practice

Six variables were used to describe the sports practice of the participants: (1) sports type (individual, team sport, both); (2) sports level (i.e., highest competition level reached; local/regional/provincial/international); (3) number of hours of practice/training/ competition per week; (4) early sports specialization, defined as an intense training in a single sport, for more than 8 months per year, before 12 years old (Laprade et al., 2016); (5) sports practice involving being away from family/parents; and (6) serious injuries over the past year, which required the expertise of a health professional.

#### Athletic behaviors

Three variables were used to measure and describe the athletic behaviors of the participants: (1) extreme weight-control behaviors (EWCB) (1 item), which referred to ever having used an extreme method (fasting, excessive exercise, use of vomiting, laxatives, diet pills, diuretics) to reach the ideal weight for their sport; (2) non-suicidal self-injury, referring to the participant ever having injured him or herself as a punishment or to motivate himself or herself (1 item); and (3) training and competing despite injury (1 item).

#### Mental health

The study included three variables related to the mental health of participants: (1) self-esteem was measured using the five items from the General Self-Esteem Scale of the Self-Description Questionnaire-II (Marsh, 1990). In this study, the scale achieved good internal consistency (Cronbach’s α = .85); (2) psychological distress was measured with the Kessler Psychological Distress Scale (10 items) (Kessler et al., 2002). This scale had been previously validated with teenagers and had good concurrent validity and internal consistency (Cronbach’s α > .92; Kessler et al., 2002). In this study, Cronbach’s α was .88.; (3) PTSD symptoms were measured using the Primary care PTSD screen (4 items) (Prins et al., 2003). This scale had good concurrent validity and good test–retest reliability (Prins et al., 2003). In this study, Cronbach’s α was .74.

### Statistical Analyses

First, LCAs were performed using PROC LCA, an add-on for SAS 9.4 for Windows (Lanza et al., 2007), to identify victimization classes. While LCA has been primarily used in the health and medical domains, it has been increasingly used in behavioral research, particularly in criminology, over the past few years (e.g., Deslauriers-Varin & Beauregard, 2010; Spaan et al., 2020). LCA assumes that discrete latent variables underlie a specific population and helps to identify underlying patterns in data or subgroups of individuals who share important characteristics or behaviors (Collins & Lanza, 2010). More specifically, LCA predicts subjects’ subgroup membership based on their responses to a set of observed categorical variables and produces mutually exclusive and exhaustive classes of individuals (Goodman, 1974; Lanza et al., 2007). LCA is particularly valuable when the theoretical construct of interest comprises of qualitatively different subgroups of individuals, but the subgroup membership of individuals is unknown and must therefore be inferred from the data (Collins & Lanza, 2010). LCA is based on two critical assumptions. First, it assumes that all individuals in a latent class have the same conditional response probabilities for the items. Second, there is an assumption of conditional independence of the latent classes identified (Lanza et al., 2007). Following LCA^2^, additional chi-square analyses were carried out, using IBM SPSS Statistics 26, between the identified classes and considered variables (e.g., sports practice, athletic behaviors, mental health) to further describe the different classes identified. Pairwise comparisons of column proportions were computed using the Bonferroni correction, which adjusts the observed significance level for the multiple comparisons.

## Results

### Identification of Latent Subgroups

LCAs were performed based on eight variables related to self-reported experiences of interpersonal violence in sport. The LCAs were inspected for two- to eight-class solutions and were repeated using 10 different sets of starting values (Lanza et al., 2007). For all information criteria used to compare solutions^3^, a smaller value for a particular model suggests that the trade-off between model fit and parsimony was achieved. After inspection, information criteria did not clearly indicate the best solution and suggested that a solution between three and six classes would be a good fit. A more thorough inspection of the parameter estimates for the three-class solution suggested that the classes found were distinguishable, non-trivial (i.e., no class with a near-zero probability of membership), and that meaningful labels could be assigned to each class found. Therefore, the three-class solution was selected as the model providing the best overall fit to the data (see Table 2).

**Table 2.** Comparison of Baseline Models.

No. of Classes	Degrees of Freedom	AIC	BIC	Adjusted BIC	Entropy
2	238	336.00	420.38	366.38	0.66
**3**	**229**	**276.44**	**405.48**	**322.40**	**0.67**
4	220	250.09	423.80	312.63	0.62
5	211	248.44	466.83	327.07	0.64
6	202	244.80	507.85	339.52	0.71
7	193	253.07	560.79	363.87	0.67
8	184	260.86	613.24	387.74	0.64

*Note.* Boldface type indicates the selected model. AIC = Akaike’s information criterion (Akaike, 1974); ABIC = adjusted Bayesian information criterion (Sclove, 1987); BIC = Bayesian information criterion (Schwarz, 1978).

The likelihood-ratio G^2^ statistic was then used to compare which three-class solution was the best (lowest G^2^ value) among the different three-class solutions obtained using the different starting values. The best-fit three-class solution selected presented a good intra-class classification accuracy based on posterior probabilities,^4^ confirming their stability and relevance. The assigned label and probability of membership for each class, as well as the item-response probabilities for endorsing each item of the class, are shown in Table 3. Item-response probabilities allow interpreting latent classes by indicating the probability of the indicators, which are conditional for class membership. Item-response probabilities vary from 0 to 1.00; an item-response probability closer to 1.00 indicates the presence of the item for the class. Item-response probabilities falling between 0.45 and 0.55 were interpreted as the somewhat arbitrary absence/presence of the item. Comparing item-response probabilities between classes therefore allows assessment of the distinctness of each identified class.

**Table 3.** Item Response for Three-Class Model Based on Probability of Endorsing Item Given Latent Class.

	Latent Classes		
Item	*Non-Victimization* 37.3% (*n* = 387)	*Psychological Victimization* 52.4% (*n* = 592)	*Poly-Victimization* 10.3% (*n* = 78)
Sexual victimization			
By teammate	0.04 (No)	0.25 (No)	0.77 (Yes)
By coach	0.01 (No)	0.14 (No)	0.45 (No)
Psychological victimization			
By teammate	0.33 (No)	0.77 (Yes)	0.98 (Yes)
By coach	0.31 (No)	0.89 (Yes)	1.00 (Yes)
By parents	0.06 (No)	0.63 (Yes)	0.87 (Yes)
Physical victimization			
By teammate	0.08 (No)	0.19 (No)	0.58 (Yes)
By coach	0.02 (No)	0.15 (No)	0.58 (Yes)
By parents	0.00 (No)	0.09 (No)	0.34 (No)

*Note:* Item-response probabilities vary from 0 to 1.00; an item-response probability closer to 1.00 indicates the presence of the item for the class, whereas an item-response probability closer to 0.00 indicate the absence of this item to define the observed class. Item-response probabilities falling between 0.45 and 0.55 were interpreted as the somewhat arbitrary absence/presence of the item and therefore cannot help to define the observed class in a meaningful way.

**Figure A1.**
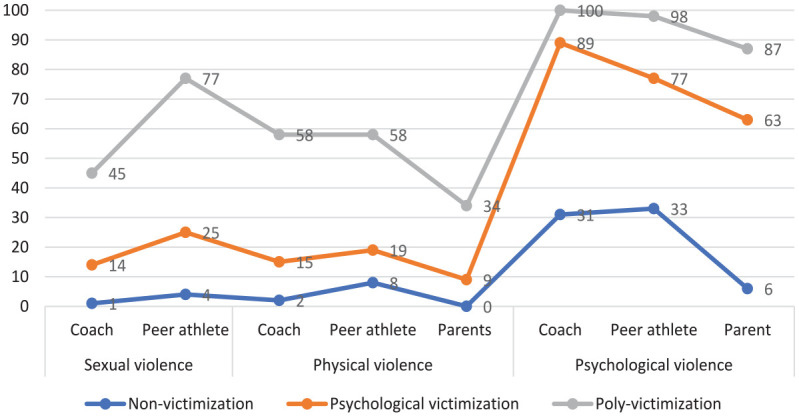
Prevalence of victimization (%) by various actors based on identified classes.

The first class/profile identified regroups young athletes that did not report being victimized in any form or by any perpetrator in a sports context (labeled the *Non-victimization* class; *NV*). This class represents close to 40% of the young athletes included in this study (37.8%, *n* = 387). The second class identified through LCA report having experienced psychological victimization at least once in the course of their sports practice (labeled the *Psychological victimization* class; *PV*). The PV class is the most prevalent one identified, regrouping more than half of the sample (52.4%, *n* = 592). Young athletes falling into this profile generally report being psychologically victimized at least once in a sports context, but by three different perpetrators: a peer (0.77), a coach (0.89), and a parent (0.63). The majority were therefore repeat psychological victims that suffered psychological victimization at the hand of different perpetrators on more than one occasion. The third and last class identified, which represents the least prevalent profile of victimization among young athletes in a sports context (10.3%, *n* = 78), regroups participants that report having experienced multiple forms of violence, perpetrated by different perpetrators (labeled the *Poly-victimization* class; *PO*). Finkelhor et al. (2007a) suggested the use of the term “life-time” or “last year” poly-victimization in reference to a subgroup of highly victimized youth within their sample. In this study, the poly-victimization profile refers to being exposed to at least three of the different forms of violence studied. Most young athletes classified under this profile have experienced at least one incident of psychological victimization by a coach (1.00), a peer athlete (0.98), or a parent (0.87); at least one incident of sexual victimization by a peer (0.77), and—in a somewhat lower proportion—at least one incident of physical victimization by a peer (0.58) or a coach (0.58). Figure A1 presents the victimization experiences of each class, based on their item-response probabilities, presented in percentages for ease of interpretation and class comparison.

### Subgroup Comparisons

To further describe the three victimization profiles identified through LCA, chi-square tests were conducted. Table 4 presents post-hoc pairwise comparisons of column proportions that were conducted, allowing determination of which pairs of columns (for a given row) are significantly different from each other, using the Bonferroni correction. Significant differences among the three victimization subgroups identified were found for all but one of the 14 variables included in the current study (i.e., type of sport).

**Table 4.** Cross-Tabulation–% (*n*)–of the Victimization Latent Classes and Variables Related to Sociodemographic, Sport Practice, Athletic Behaviors and Mental Health of Participants.

	Victimization Profiles			
	NV (*n* = 387; 37%)	PV (*n* = 592; 52%)	PO (*n* = 78; 10%)	Subgroup Comparisons
Sociodemographic				
Age (*n* = 1,057)	15.13 (SD = 1.05)^b c^	15.29 (SD = 1.06)	15.46 (SD = 1.10)	F(2, 7.73) = 6.86**
Gender (*n* = 1,056)				
Boy	32.3% (125)	23.8 (141)^a^	33.8 (26)	χ^2^(2) = 9.97**
Girl	67.7% (262)	76.2 (451)^c^	66.2 (51)	Cramer’s V = 0.10
Sport practice				
Sport type (*n* = 1,050)				
Individual	44.6% (172)	42.2% (247)	35.9% (28)	χ^2^(4) = 5.18, NS
Team	50.0% (193)	54.1% (317)	61.5% (48)	
Both	5.4% (21)	3.8% (22)	2.6% (2)	
Sport level (*n* = 1,030)				
Local/Regional/Interregional	30.4%(116)^b^	23.8% (138)	28.6% (22)	χ^2^(4) = 10.26*
Provincial	46.5% (177)	46.8% (272)	53.2% (41)	Cramer’s V = 0.07
National/International	23.1% (88)	29.4%(171)^a c^	18.2% (14)	
Weekly hours of practice (*n* = 1,046)				
Less than 5 hours	16.0% (61)	13.5% (79)	11.7% (9)	χ^2^(6) = 26.67***
6 to 10 hours	44.8%(171)^b^	33.6% (197)	33.8% (26)	Cramer’s V = 0.11
11 to 15 hours	25.4% (97)	27.3% (160)	26.0% (20)	
16 and up	13.9% (53)^b c^	25.7% (151)	28.6% (22)	
Early sport specialization—Yes (*n* = 1,057)	20.7% (80)	24.8% (147)	38.5% (30)^a b^	χ^2^(2) = 11.36**
				Cramer’s V = 0.10
Training away from parents—Yes (*n* = 1,029)	5.0% (19)	6.6% (38)	16.4% (12)^a b^	χ^2^(2) = 12.75**
				Cramer’s V = 0.11
Serious injury—Yes (*n* = 977)	62.9% (220)^b c^	79.2% (443)	83.8% (57)	χ^2^(2) = 33.70***
				Cramer’s V = 0.19
Athletic behaviors				
Extreme weight-control behaviors—Yes (*n* = 978)	7.4% (26)^b c^	20.7% (116)	32.4% (22)	χ^2^(2) = 39.96***
				Cramer’s V = 0.20
Non-suicidal self-injury—Yes (*n* = 978)	3.7% (13)^b c^	20.0% (112)^a c^	54.4% (37)^a b^	χ^2^(2) = 117.08***
				Cramer’s V = 0.35
Practice despite injuries—Yes (*n* = 978)	32.3% (113)^b c^	67.0% (375)^a c^	83.8% (57)^a b^	χ^2^(2) = 128.37***
				Cramer’s V = 0.36
Mental Health				
Self-esteem (*n* = 969)				
Normal	76.7% (267)^b c^	60.4% (334)^a c^	44.1% (30)^a b^	χ^2^(2) = 39.26***
Low	23.3% (81)^b c^	39.6% (219)^a c^	55.9% (38)^a b^	Cramer’s V = 0.20
Psychological distress (*n* = 965)				
Low	64.3% (222)^b c^	37.0% (204)^a c^	10.3% (7) ^a b^	χ^2^(4) = 124.92***
Moderate	20.9% (72)	27.2% (150)	20.6% (14)	
Severe	14.8% (51)^b c^	35.9% (198)^a c^	69.1% (47)^a b^	Cramer’s V = 0.25
PTSD symptoms—Yes (n = 958)	19.5% (67) ^b c^	44.4% (243)^a c^	66.2% (45)^a b^	χ^2^(2) = 82.61***
				Cramer’s V = 0.29

*Note:* NV = non-victimization; PV = psychological victimization; PO = poly-victimization; PTSD = post-traumatic stress disorder; SD = standard deviation.

a Different from NV class.

b Different from PV class.

c Different from PO class.

* *p* < .05. **p < .01. ***p < .001.

#### Sociodemographic characteristics

Significant subgroups mean differences were found for both age and gender (*p* < .01). The *NV* subgroup was younger (Mean [*M*] = 15.13 years; SD = 1.05) than both the *PV* (*M* = 15.29; SD = 1.06) and *PO* subgroups (*M* = 15.46; SD = 1.10). Furthermore, the *PV* subgroup was found to be composed of a significantly higher proportion of girls (76%) compared to the *NV* subgroup (68%).

#### Sports practice and athletic behaviors

The subgroups identified showed significant differences in their sports practice and athletic behaviors. More specifically, subgroup analyses showed that (1) the *PV* subgroup included a higher proportion (*p* < .05) of young athletes practicing sports at a higher competitive level (National/International; 29%) than the *NV* (23%) or the *PO* (18%) subgroups; (2) athletes in the *NV* subgroup were more likely (*p* < 0.001) than the other subgroups to practice between 6 and 10 hours weekly (45% vs. ≈34%) and less likely to weekly spend 16 hours or more practicing their sport (14% vs. 26% and 29%); (3) athletes in the *PO* subgroup were more likely (*p* < .01) to show an early sports specialization (38%) that the other subgroups (21%, 25%), and (4) to report practicing a sport that kept them away from their parents and family (16% vs. 5% and 7%); and (5) the *NV* subgroup was less likely than the other two subgroups to report having been seriously injured over the past year (63% vs. 79% and 84%). Finally, EWCB, non-suicidal self-injuries, and practicing despite injuries were less likely observed in the *NV* subgroup compared to the *PV* and *PO* subgroups (*NV*<*PV*<*PO*).

#### Mental health

Significant differences were found for the three mental health issues between the three victimization subgroups identified. The *NV* subgroup reported fewer problems in low self-esteem (23% vs. 40% vs. 56%), severe psychological distress (15% vs. 36% vs. 69%), and a clinical threshold for PTSD (19% vs. 44% vs. 66%) compared to the *PV* and the *PO* subgroups (*NV* < *PV* < *PO*).

**Figure A2.**
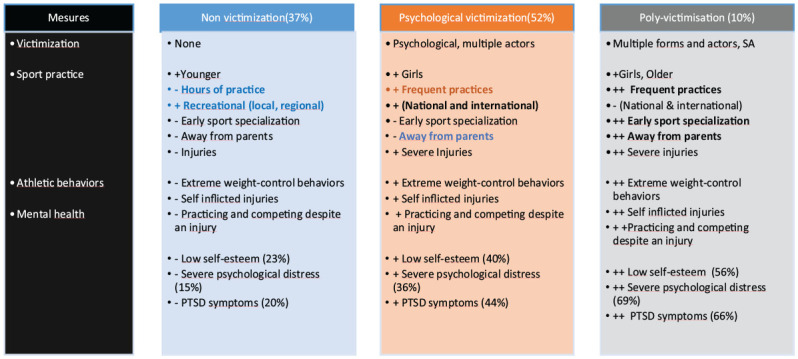
Summary of the class comparisons on the sport practice, athletic behaviors, and mental health characteristics.

Figure A2 proposes an overall summary of the comparisons between classes. In short, athletes in the *NV* subgroup were more likely to be younger, practice a sport at a somewhat less competitive level (local/regional/interregional), and spend a lesser number of hours per week practicing their sport, while being less likely to report serious injuries in the past year and present problematic athletic behaviors and mental health issues. Athletes assigned to the *PV* subgroup were more likely to be girls, compete at a higher level (national/international), while presenting an increased probability of problematic athletic behaviors and mental issues (compared to the *NV* subgroup). Finally, athletes in the *PO* subgroup were more likely to show an early sports specialization and practicing a sport that kept them away from their parents, while also being the subgroup showing the highest (and really concerning) proportions of various problematic athletic behaviors and mental health issues.

## Discussion

Interpersonal violence makes its way into the sport context and generates numerous undesirable outcomes (Mountjoy et al., 2016). How are victimization classes useful to this field? If somewhere between 2% and 75% of athletes are confronted to this issue (Parent & Vaillancourt-Morel, 2020; Vertommen et al., 2016), and with poly-victimization being a concern (Vertommen et al., 2018), the person-oriented approach is useful for it avoids grouping together victimized individuals with extremely different experiences and needs. While this section reviews each of the victimization profile’s level of risk, protection, and related outcomes, the discussion is organized around three general findings: (1) the high prevalence of psychological violence in sport and the intense and high level of sports practice in which it appears more likely to develop, (2) the all-embracing outcomes related to multiple or poly-victimization, and (3) contexts associated to the protection of athletes.

### High Prevalence of Psychological Violence

The first remarkable observation is that one athlete out of two (52% of the sample) classifies within the psychological, *PV* profile by various perpetrators. Of particular interest is that athletes within this class are most likely of all to practice their sport at the national or international level; and as for the *PO* class, to train more than 16 hours a week. As discussed in prior studies (i.e., Mountjoy et al., 2016; Parent & Fortier, 2018), these two elements appear to represent risk factors for violence exposure. On the other hand, the sports practice of teenagers within the *PV* class differs from the *PO* class, as the *PV* class benefits from the regular presence of parents, even if 30% of these athletes are performing at the national or international levels. Aside from psychological violence, the regular presence of parents thus appears to provide protection from exposure to other forms of violence by peers or coaches. This finding supports the Routine Activities Theory (Cohen & Felson, 1979) according to which victimization tends to occur more frequently in situations where (a) there is a lack of guardianship (in this case parents of friends), (b) if a suitable target is available (or in this case vulnerable, or willing to tolerate), and (c) in the presence of motivated “offenders” (in this case coaches, parents and peers who are often isolated within this context of high performance). For the *PV* class, although athletes experience psychological violence by peers, coaches, and athletes, the presence of parents may protect from additional sexual and physical violence. Nonetheless, the related outcomes to violence exposure within this class (i.e., *PV*) are worrisome, in particular for athletic behaviors that are more alike to the *PO* than to the *NV* class. In fact, up to 67% of the *PV* class report training and competing despite an injury, which is more than twice the prevalence observed within the *NV* class. EWCB and self-injury are also more frequent within this class and within the *PO* class than in the *NV* class. Further, more than a third of these athletes that were exposed to psychological violence report low self-esteem, PTSD symptoms and high levels of psychological distress. If psychological violence is rather prevalent within the entire sample (between 44% and 68%); its related consequences within this class clearly demonstrate the importance of preventing its occurrence for athletes.

### Poly-victimization and Its Related Outcomes

The second noteworthy finding is the generalized impact associated with poly-victimization in the young athlete’s life. Two elements of risk clearly distinguish the *PO* class from the others: sports practice away from parents and, as hypothesized by Malina (2010), early sports specialization. In addition to these conditions, athletes within this class also share two other factors with the *PV* class, which may represent additional or cumulative risk: practicing their sport more than 16 hours a week and at higher levels than the regional/local level. Another particularity of this class is that despite many hours of sports practice away from parents and early sports specialization, which may, at first glance, be described as a context associated with higher performance, athletes in the *PO* class are significantly less likely than the other classes to practice their sport at the national or international level. The poly-victimized class is characterized by a compromised mental health and problematic athletic behaviors, which may explain that, although these youth are particularly invested in their sport, they do not yet appear to reach the elite level but are close to reaching it. Brackenridge and Kirby (1997) proposed the concept of “stage of imminent achievement” (SIA), which refers to the period during which an athlete has not yet reached peak performance but performs very close to that level. Authors argue that during adolescence, as youth become sexually active and enter the ages at which sports careers are likely to peak, it is thought that this “SIA” may represent a period of particularly acute vulnerability to sexual abuse. During this period, the thought of dropping out when faced with adversity (i.e., sexual abuse) may appear more costly for those who are close to the elite level. In line with Ohlert’s German study exploring the victimization of elite athletes (2019), our study reveals that the *PO* class presents with the highest prevalence of mental health problems (self-esteem, psychological distress, and PTSD) and problematic athletic behaviors. They are more likely than the *NV* class to have experienced a serious injury, to suffer from eating disorders and, most likely of all classes, to suffer from non-suicidal self-injury or to practice their sport despite an injury, emphasizing the importance of early intervention.

### Contexts Associated With the Protection of Athletes

The results of this study regarding the *NV* class are particularly interesting as teenagers constituting this subgroup appear to benefit from a certain number of factors that may be interpreted as protective. Whether adolescents practiced individual, or team sports does not influence violence exposure. On the one hand, youth in the *NV* class are younger, they are practicing less regularly than the other subgroups, and mostly at the local or regional level. On the other hand, the sports practice of these non-victimized teenagers can also be described as more recreational and less specialized than the *PO* class, as indicated by a significantly lower percentage of teenagers engaged in early sports specialization and of teenagers practicing their sport away from parents. As such, these teenagers are less often injured and less likely to pursue their sport practice despite an injury, and a fewer of these youth are reporting low self-esteem, psychological distress, and PTSD.

Taken together, the distribution of victimization profiles observed in this study has been observed in other studies conducted with various samples of youth and adolescents in and outside of the sport context; where a subgroup is usually exempt from victimization, while another is overly exposed, and the others are found somewhere in the middle of those two extremes (Davis et al., 2019; Vertommen et al., 2018). As outlined by researchers, the most relevant information associated with these profiles are the rather consistent observations that multiple victimizations (e.g., *PV* class) and multiple forms of victimization (e.g., *PO* class) are associated with important mental health outcomes and problematic behaviors (Butcher et al., 2016; Davis et al., 2019). Our results indicate that such observations also apply to the sport context where the various profiles of victimization are differentially associated with problematic sports practice patterns and athletic behaviors. Thus, concordant with the findings of Vertommen et al. (2018), the results of this study can be conceptualized along a continuum of victimization with at risk athletic behaviors and mental health outcomes proportionally increasing with reported exposure to violence. Results also reinforce the idea that there are poly-victims in the sport context and, most troubling, that they are being poly-victimized within that specific context. Although our results do not allow to account for violence outside of the sport context, or for other risk factors documented in more diverse populations (e.g., minority groups) chances are that these youths are also exposed to violence in other environments (school, home) (Finkelhor et al., 2007b).

### Limitations, Future Research, and Practical Implications

This study documented violence exposure within a sample representing French Canadian youth who are practicing different sports (individual, team) in various contexts (grassroot to elite level). While results concern a large variety of Caucasian youth practicing sport, the sample representation of specific ethnic and minority groups was insufficient to integrate various forms of diversity. Larger samples could allow to further explore risk exposure and related outcomes among diverse culture, racial, ethnic, and minority groups. Furthermore, the associated outcomes to violence exposure did not consider the severity or duration, nor controlled for violence exposure outside of sport (home or school). Results suggest that future research should further explore the combined presence of early sports specialization and intense sports practice, which appear to act as a risk factor for exposure to violence. Based on our findings, if children begin sports practice early in their development (US National Council of Youth Sports, 2008) and especially in a context of early sports specialization, they could benefit, along with their parents and coaches, from primary prevention measures addressing healthy and safe sports practice, to compensate from this potential risk of violence exposure.

Along with other studies (e.g., Vertommen et al., 2018), our findings also suggest that youth practicing sport intensively in a high-performance context (i.e., many hours per week, early sports specialization profile, elite athletes) may present higher risks to violence exposure. As discussed, the *PV* class is composed of a large percentage of elite athletes, whereas individuals in the *PO* class are more likely to fit with an early sports specialization profile. These contexts of sports practice are largely influenced by a desire to maintain high standards of performance to be, or to become the best (Parent & Fortier, 2018). In this context, athletes, parents, and coaches may be more likely to accept compromises and sacrifices (i.e., living away from parents, in proximity with coaches and other athletes, training more than 16 hours a week) and to endure some form of physical and/or psychological discomfort (Cense & Brackenridge, 2001; David, 2004). These particular contexts of sport practice may require regular monitoring and specific preventive and educational measures to promote early identification of violence and of its related negative behavioral, mental, and physical health outcomes, that may hinder long-term well-being.

## Conclusion

Within our daily occupations, it is widely recognized that the practice of sports is intended to bring pleasure, health, and well-being. Somehow, along with standards of performance, devotion, compromise, and tolerance, violence appears to have made its way to various contexts of sport practice. Current and past research suggests that raising public awareness on the insidious occurrence of both overt and covert forms of violence should be a priority. Stakeholders, administrators, coaching staff, athletes, parents, and spectators need to be better informed of these occurrences and of its related consequences. Education and training deserve to be invested for all actors within the sport environment so that channels of communication are clearly identified, publicly recognized, and open. The profiles identified in this study suggest that level of exposure to single and multiple forms of violence is proportionately related to mental health outcomes and problematic sport and athletic behaviors. Such results outline the need for prevention, early detection, and appropriate intervention to prevent such short- and long-term effects and to promote the protection of athletes and the pleasure and health benefits associated with sport.

## Notes

1. Spearman’s Rho correlations were conducted between all variables to be included in the LCA models prior to the LCA analyses to test for collinearity issues. No issue was detected; Spearman’s rho were all <0.45.

2. For more information about the participants, procedures, and measures used in this study, see the original study (Parent et al., 2016)

3. The Bayesian information criterion (BIC; Schwarz, 1978), Akaike’s information criterion (AIC; Akaike, 1974), and adjusted Bayesian information criterion (ABIC; Sclove, 1987) are penalized log-likelihood model information criteria that were used to compare competing model fit to the same data (i.e., models with different numbers of latent classes). It was decided to also use the ABIC to better identify the best-fit model considering that the BIC sometimes tends to underestimate the number of latent classes (Yang, 2006).

4. Average assignment probabilities based on posterior probabilities for the three-class solution: Class 1 (*Non-victimization;* NV) = 0.88 (0.53–0.99); Class 2 (*Psychological victimization;* PV) = 0.83 (0.54–1.00); and Class 3 (*Poly-victimization;* PO) = 0.82 (0.51–0.99).
